# Salivary gland proteins alterations in the diabetic milieu

**DOI:** 10.1007/s10735-021-09999-5

**Published:** 2021-07-01

**Authors:** Malak Fouani, Charbel A. Basset, Abdo R. Jurjus, Lavinia Giovanna Leone, Giovanni Tomasello, Angelo Leone

**Affiliations:** 1 Department of Biomedicine, Neuroscience and Advanced Diagnostics, Institute of Human Anatomy and Histology, University of Palermo, Palermo, Italy; 2 Department of Anatomy, Biology and Physiological Sciences, Faculty of Medicine, American University of Beirut, Beirut, Lebanon; 3 School of Medicine, University of Palermo, Palermo, Italy

**Keywords:** Salivary glands, Diabetes mellitus, Muscarinic receptors, Biomarkers, Screening

## Abstract

Salivary glands are considered the chief exocrine glands of the mouth and physiologically contribute to the maintenance of the homeostasis of the oral cavity. They consist of the parotid, submandibular and sublingual glands, which come in pairs and are collectively called the major glands, and the minor glands, which are much smaller and are dispersed throughout the buccal cavity. Salivary glands are distinguished by their size, amount of saliva secretion and their location in the oral cavity. Salivary glands pathophysiology has been a subject of interest in various worldwide metabolic disorders, including diabetes mellitus. Diabetes mellitus (DM), a global health concern, with a pathological imprint involved in vasculature, promotes microvascular and macrovascular complications among which periodontitis ranks sixth. Indeed, DM has also been directly associated with oral health lesions. Specifically, salivary glands in the context of diabetes have been a focal point of study and emphasis in the research field. There is evidence that relates salivary secretion content and diabetes progression. In this review, we present all the reported evidence of the deregulation of specific salivary proteins associated with the progression of diabetes in parallel with changes in salivary gland morphology, cellular architecture, and salivary secretion and composition more generally.

## Introduction

Anatomically, the salivary glands constitute a system of exocrine glands localized in the head, in and around the buccal cavity. Under physiological conditions, salivary contents are released through a system of ducts into the mouth. Salivary gland secretions contribute to oral health by keeping the oral mucosa protected and lubricated (Contreras-Aguilar and Gómez-García 2020). They are also responsible for initiating early stages of digestion in the oral cavity by two mechanisms: (1) assisting in the creation of a food bolus during the mastication procedure thus allowing its swallowing for further processing; (2) through their enzymatic content, namely alpha-amylase, which breaks down starch into maltose and glucose. The glands are differentiated by their size and are classified based on the type of the saliva they excrete (Basset et al. 2020a). Diabetes Mellitus (DM) is a growing global health concern that impacts the global health expenditure. DM is classified into type 1 (T1DM) and type 2 diabetes (T2DM). The former is referred to as “juvenile” diabetes in which it occurs at a young age and is mediated by an autoimmune destruction of the insulin-producing β cells of the pancreas; while the latter pertains to cellular insulin resistance and is statistically prevalent (Kudiyirickal and Pappachan 2015). DM is usually accompanied by a plethora of complications, including oral health diseases (Saini et al. 2011; Zhang et al. 2014). Recently, a body of evidence has shed light on the importance of oral complications in DM patients and underlined their interconnection (Kudiyirickal and Pappachan 2015). In this review, we present findings that underpin the correlation between DM and salivary gland complications, specifically the alterations of key proteins in diabetic patients that gravely moderate the function of salivary glands and ultimately impact patients daily lifestyle.

## Salivary gland diseases and Diabetes Mellitus

### Salivary Glands physiology and anatomy

The salivary glands are classified into two main groups: minor and major. The major glands are much larger in size and account for most of the salivary secretions, a function directly linked to their histology and morphology as they consist of a collection of exocrine tissues that secrete as a whole into a salivary duct rather than acting individually. Minor and major salivary glands not only differ by the amount of saliva they produce but also by the quality of salivary secretions. Minor gland saliva serves to lubricate the walls of the oral cavity, while major gland saliva is digestive and protective in nature. There are three pairs of major salivary glands that open into the mouth through distinctive ducts. The parotid salivary glands, the largest of the three, are enclosed in a tissue capsule and are composed of fat tissue and cells. They mainly produce serous fluids which account for approximately 25–30% of the total daily salivary output. Each gland’s main duct, called Stensen’s duct, opens in the rear of the mouth cavity near the second upper molar. The second pair, the submandibular glands, are located along the side of the lower jawbone, these glands are surrounded by a capsule of tissue which give off mixed secretions mostly serous in nature. It secretes by far the largest volume of saliva of all roughly 70% of the total daily output. The Wharton’s duct of each of those glands opens into the floor of the mouth at the junction where the front of the tongue meets the mouth’s floor. The third pair are the smallest in size, the sublingual glands, located beneath the mucous membrane of the floor of the mouth, near the chin region. These are dispersed throughout the surrounding tissues since they are not encapsulated as the aforementioned glands. Their numerous Rivinus’s ducts empty near the junction of the tongue and the mouth’s floor while some of them fuse to form Bartholin’s duct, the main duct of the sublingual gland, which discharges into the submaxillary duct. These glands secrete a mixed fluid that is mainly mucus which accounts for only 5% of the total daily salivary output (Holmberg and Hoffman 2014; Kessler and Bhatt 2018).

Minor salivary gland tissue is approximately comprised of 800–1000 small salivary glands dispersed as patches throughout the submucosa of the sino nasal cavity, oral cavity, pharynx, larynx, trachea, lungs, and middle ear cavity. Minor salivary glands produce roughly 1% or less of the total salivary output. Although minor salivary gland tissue can be found anywhere along the aerodigestive tract, it is most condensed along the labial mucosa, lingual mucosa, buccal mucosa, the soft palate, the lateral parts of the hard palate, and the floor of the mouth (Holmberg and Hoffman 2014).

Saliva has several major functions within the oral cavity which involve buffering, lubrication, antimicrobial job, digestion, hormone regulation and taste sensation. Saliva is composed of 99.5% water and 0.5% of glycoproteins, mucus, electrolytes, enzymes and antibacterial composites. Humans produce between 0.5 to 1.5 L of saliva every day. This secretion is initiated by parasympathetic stimulation; the neurotransmitter activated in this process is acetylcholine in which it binds to the muscarinic receptors in the gland, leading to amplified salivation (Holmberg and Hoffman 2014). The process in which saliva is formed is composed of two phases: (1) During phase one, salivary secretions are formed in the acinar cells (serous and/or mucus cells) within the rough endoplasmic reticulum by ribosomal protein synthesis and are stored as granules. These granules are later discharged into the lumen by exocytosis. (2) In the second phase, the saliva is subdued to chemical changes while passing through the salivary ductal system as it reaches the oral cavity. As it reaches the intercalated ducts, isotonic saliva secreted by the acini is mixed with lysozymes and lactoferrin produced by the intercalated duct cells. In the striated ducts, reabsorption of chloride and sodium takes place concomitantly with kallikrein and epidermal growth factor release and since these ducts are impermeable to water, the final outcome is a hypotonic state of saliva ready to be released into the oral cavity through the excretory ducts (Romanenko et al. 2008; Punj 2019; Contreras-Aguilar and Gómez-García 2020). Myoepithelial cells participate in the flow and secretion of saliva by providing contractile support to the acinar cells and the intercalated ducts (Proctor 2016). Through secretions and exocytosis-mediated release by the acinar cells as well as the flow of saliva into the ducts, salivary proteins such as proline-rich proteins, histatins, cystatin, defensins, cathelicidin-LL37, and enzymes such as amylase (ptyalin), peroxidase and lysozyme are being released and mixed into the saliva constituting the bulk of salivary proteins (Proctor 2016).

### Diabetes and oral manifestations are intertwined

As mentioned previously, DM is usually accompanied by a plethora of complications, including oral health diseases (Kudiyirickal and Pappachan 2015). Periodontitis was ranked sixth among those complications (Saini et al. 2011). DM and periodontal complications have a mutually reinforcing effect on each other: DM increases the ruthlessness, occurrence and progression of periodontal disease while periodontal disease may accentuate the severity of DM by deteriorating the level of glycemic control in the blood (Meenawat et al. 2013). Moreover, studies have shown that periodontal inflammation is significantly increased in subjects with longer disease course, poor metabolic control (Arana et al. 2017) and DM complications (Preshaw et al. 2012). This makes it more dominant in T1DM patients whereas T2DM are more prone to develop tumors at different sites, including the salivary glands (Suba et al. 2005). This is in contrast with xerostomia (hyposalivation) that has been shown to be prominent in both types of DM (Hoseini et al. 2017).

### Salivary gland diseases in diabetes

A plethora of disease processes can occur within salivary gland tissue. Their prevalence depends on various aetiological factors. Common infectious/inflammatory processes within the major and minor salivary glands have been reported. These may include bacterial, viral infections, rarely fungal and ductal obstruction which may cause agonizing swelling or blockage, affecting their function (Kessler and Bhatt 2018). Although scarce, salivary glands may also be affected of benign and malignant tumors classified under 31 different types (El-Naggar et al. 2017). In addition, recent research has shed light on the strong association between salivary gland diseases and DM, a major growing public health concern as one of the leading causes of mortality and morbidity globally (Rohani 2019).

DM is associated with enhanced periodontal tissue destruction, which requires from DM patients an extreme management of periodontal disease and rapid control of ongoing infections to avoid exacerbation of the existing metabolic imbalance (Rohani 2019). On the cellular and molecular levels, particularly, the oral cavity and associated structures remains a somewhat under‐examined area in diabetes mellitus research. Studying salivary gland dysfunction markers and protein expression in DM patients may hold a great potential for increasing our understanding of the pathophysiology of this multifactorial disease. Using proteomics-based technologies for detecting salivary gland hallmark proteins in diabetic patients is definitely a promising tool for detecting pathological abnormalities prior to development of clinical symptoms.

### The role of oxidative stress in diabetes-related oral disease

Chronic oxidative stress is a state that occurs in DM due to persistent hyperglycemia, which stimulates a sequence of mechanisms that generate excessive production of reactive oxygen species (ROS). In addition, it has been well established that ROS overproduction is involved in mediating a state of oxidative stress being the final pathway responsible for the onset and progression of DM complications (Giacco and Brownlee 2010). Taking in account the importance of oxidative stress in driving the onset and progression of DM complications, and periodontitis being ranked sixth among DM complications (Saini et al. 2011), we ought to pinpoint its importance in diabetes-related oral manifestations. ROS are highly reactive chemical molecules that when increased dramatically under stressful conditions lead to significant damage of cellular structures especially in the case of weakened antioxidant systems (Knaś et al. 2016). Different oxidative stress markers such as 8-Hydroxy-2′-deoxyguanosine (8-OHdG), protein carbonyl (PC), 4-hydroxynonenal protein adduct (4-HNE), oxidized and/or MDA-modified LDL-cholesterol (oxy-LDL/MDA) and 8-isoprostanes (8-isoP) were measured in streptozotocin (STZ)-induced DM rats (Knaś et al. 2016). Furthermore, this study evaluated oxidative damage caused to the salivary glands in STZ-induced DM. Results showed the three major salivary glands undergo increased oxidative stress in STZ rats regardless of the duration of the disease. However, the parotid glands seemed more vulnerable showing greater extent and diversity of oxidative injury in this gland. Moreover, decrease of the stimulated saliva secretion is detected in the first week of the experiment. In the progressive stages of the disease, simulated saliva secretion is significantly more reduced than the secretion of non-stimulated saliva (Knaś et al. 2016). Another study has shown similar results pertaining to the role of oxidative stress (OS) in the pathogenesis of the salivary gland dysfunction in the course of insulin resistance (IR) in high fat diet (HFD) fed rats (Kolodziej et al. 2017). Results revealed increased oxidative stress markers in IR rats compared to control as well as significant decrease in stimulated salivary flow rate. But most importantly, IR is a prediabetic state and subsequently, salivary gland dysfunctions could be identified early in the progressive course of this disease and could be also detected in prediabetic state. Thus, IR should be taken seriously in dental clinics as at this stage of DM it could lead to oral health manifestations (Kolodziej et al. 2017). Oxidative stress marker, oxidized glutathione/reduced glutathione (GSSG/GSH), was shown to be increased in the saliva of T2DM patients compared to control group (Arana et al. 2006, 2017). Additionally, a significant increase in oxidative stress levels was found in the T2DM group with poor metabolic control vs the T2DM group with good metabolic control. This increase in oxidative stress was positively correlated with poor periodontal health as the Community Periodontal Index (CPI) was the highest in T2DM group with poor metabolic control as compared with T2DM group with good metabolic control and the control group respectively (Arana et al. 2017). An increase in total antioxidant power (TAOP) was reported in the saliva of T1DM patients compared to the control (Astaneie et al. 2005). A similar study reported the correlation between the increase in salivary and serum total antioxidant status (TAS), superoxide dismutase (SOD) and oxidative stress with the severity of DM/HbA1c values (Reznick et al. 2006). On the other hand, a decrease in salivary superoxide dismutase (SOD) of T1DM patients was presented as a possible driving factor of oral complications of DM (Belce et al. 2000). Taken together, these data encourage the evaluation of alterations of oxidative parameters in saliva of DM patients as it may be a non-invasive alternative for achieving a better understanding of the pathogenesis of the disease.

### Deregulation of Salivary gland protein expression in diabetes mellitus

#### Salivary amylase protein alteration in Diabetes

Alterations in salivary parameters of DM patients have been associated with changes in their metabolism afflicted by DM. One of the most studied salivary parameters is salivary amylase protein. Salivary amylase protein is a prominent protein found in the oral cavity (Pérez-Ros et al. 2021). Lately, salivary proteins as potential markers for diagnosis and prognosis of chronic disease have been a hot topic of research as they offer various advantages due to their ease of collection, less risks of infections, lower cost and mainly their non-invasive nature. In this context, salivary amylase levels have been studied in DM patients. A significant increase in salivary amylase in DM patients compared to controls under fasting (López et al. 2003; Aydin 2007; Abd-Elraheem et al. 2017) and non-fasting (Malathi et al. 2013; Priya et al. 2016; Lima-Aragão et al. 2016; Lodgotra et al. 2016; Tiongco et al. 2019; Kheirmand Parizi et al. 2019) conditions has been reported; albeit other scientist groups have shown different outcomes (Tenovuo et al. 1986; Newrick et al. 1991; Panchbhai et al. 2010; Prathibha et al. 2013; Indira et al. 2015), Table 1. Results are also rather conflicting when it comes to the correlation between blood and salivary glucose/amylase levels (Panchbhai et al. 2010; Siddiqui et al. 2015; Abd-Elraheem et al. 2017; Tiongco et al. 2019). Salivary amylase levels are certainly worth further investigations in DM patients, however for the time being, no decisive conclusion can be elaborated about their use as a substitute marker to blood glucose for screening DM patients.

**Table 1:** Examples of studies in which salivary proteins have been measured in DM

Altered protein	Location	Expression level	A/D	Salivary secretion/flow	Diabetic model	References
Amylase	Saliva	Increase	N.A	Decrease	T1DM and T2DM patients	López et al. (2003), Aydin (2007), Malathi et al. (2013), Priya et al. (2016), Lima-Aragão et al. (2016), Lodgotra et al. (2016), Abd-Elraheem et al. (2017), Tiongco et al. (2019), and Kheirmand Parizi et al. (2019)
		Decrease				Tenovuo et al. (1986), Newrick et al. (1991), Panchbhai et al. (2010), Prathibha et al. (2013), and Indira et al. (2015)
SGLT1	SMG	Increase	N.A	Decrease	T1DM Alloxan Rats (I.V)	Sabino-Silva et al. (2013)
	PG					
NOS-BH4	SMG	Decrease	Yes	Decrease	T1DM STZ Rats	Stewart et al. (2016)
	PG					
BMP7	SMG	N.S	No	Decrease	T1DM STZ Rats	Izumi et al. (2008)
	PG	Decrease	Yes			
CSP1	Serum	Increase	N.A	N.A	DM patients^a^	Wang et al. (2016)
	Saliva					Zhang et al. (2020)
Statherin	Saliva	Decrease	N.A	N.A	T1DM patients	Caseiro et al. (2013)
	PG				T2DM patients	Isola et al. (2012)
	SMG					Isola et al. (2011b)
	LG					Isola et al. (2011a)
AQP1	SMG	Decrease	Yes	Decrease	T1DM STZ Rats	Cui et al. (2021)
AQP5	SMG-PG					Wang et al. (2011), Bhattarai et al. (2017), Biswas et al. (2018), Cui et al. (2021)
AQP8	SMG					Cui et al. (2021)
M3R	SMG	Decrease	Yes	Decrease	T1DM STZ Rats	Watanabe et al. (2001)
	PG					
HSP60	Saliva	Increase	N.A	N.A	T2DM patients	Yuan et al. (2011)
	Serum					
EGF	Fetal SMG	Decrease	Yes	Decrease	T1DM STZ Rats	El Sadik et al. (2018)
CK5						
CK7						
AQP5						
Bcl2						
PCNA						
Bax		Increase				
Casp3						

*A/D* Atrophy and degeneration of salivary glands, *N.A.* not available, *T1DM* type 1 diabetes mellitus, *T2DM* type 2 diabetes mellitus, *SGLT1* sodium-glucose cotransporter 1, *SMG* submandibular glands, *PG* parotid glands, *I.V* intravenous, *NOS-BH4* nitric oxide synthase-tetrahydrobiopterin, *STZ* streptozotocin, *BMP7* bone morphogenetic protein 7, *CSP1* common salivary protein 1, *LG* labial glands, *AQP* aquaporin, *M3R* muscarinic M3 receptor, *HSP60* heat shock protein 60, *EGF* epidermal growth factor, *CK5* cytokeratin 5, *CK7* cytokeratin 7, *Bcl2* B cell lymphoma 2, *PCNA* proliferating cell nuclear antigen, *Bax* Bcl2-associated X protein, *Casp3* caspase-3

^a^Type of diabetes not specified

#### Sodium-glucose cotransporter 1 (SGLT1)

In recent studies it has been found that levels of fasting and postprandial blood glucose are drastically higher among DM patients with oral manifestations compared to those without, indicating inadequately controlled glycemic status, therefore demanding earlier evaluation of oral manifestations to detect long-term complications (Bajaj et al. 2012). Due to major association between DM patients with oral manifestations and other diseases such as retinopathy, hypertension, peripheral and autonomic neuropathy, deregulation of specific common proteins expression has been investigated. A prominent example is the high level of sodium-glucose cotransporter 1 (SGLT1) protein found in diabetic and hypertensive patients which plays a key role in xerostomia through salivary water reabsorption in salivary ducts (Sabino-Silva et al. 2013), Table 1. SGLT1 was mostly increased in the luminal membrane of ductal cells and was inversely proportional to salivary flow (Sabino-Silva et al. 2013). These findings could play a pivotal role for future studies involving SGLT1 inhibitors-based treatments for xerostomia in diabetic and hypertensive patients.

#### Nitric oxide synthase and tetrahydrobiopterin Protein (NOS-BH4)

Nitric oxide (NO), a major player in healthy salivary glands physiology, is synthesized by nitric oxide synthase (NOS) which comes in neuronal (nNOS) and endothelial (eNOS) forms. NOS is typically found dispersed in the neural terminals within the salivary glands and also in the ductile system which contains nNOS in the apical membrane of the excretory and striated ducts, the cytoplasm of granular convoluted tubules and in the cytoplasm of excretory and striated ducts (Lomniczi et al. 1998). NO’s role in salivary secretion consists in the regulation of cGMP production. Upon salivary gland muscarinic receptor activation, intracellular calcium increases and stimulates the production of NOS, that catalyzes the formation of NO, ultimately leading to the generation of cyclic guanosine monophosphate cGMP which in turn opens ion channels (Putney 1983; Dawson et al. 2001) to initiate the secretory process (Sakai et al. 2002). NOS activity was found to be crucial for the regulation of aquaporin 5 (AQP5), a major protein involved in the salivary secretion (Lasisi et al. 2019). NOS requires tetrahydrobiopterin (BH4), a cofactor of NOS, as its catalytic activity depends on a BH4-dependent dimerization (Förstermann and Sessa 2012). DM rat submandibular eNOS and nNOS protein expression as well as their enzymatic activity were shown to be decreased. BH4 was reported to be downregulated in DM rat submandibular glands and its decrease directly correlated with the degree of dimerization of NOS (Stewart et al. 2016), Table 1. Consequently, BH4 insufficiency leads to n/e NOS uncoupling and reduced enzymatic activity; n/e NOS activity represents a critical signaling knot for adjusting salivary gland function (Stewart et al. 2016). These data encourage research to determine the mechanisms underpinning the decrease of NOS-BH4 protein expression as a trigger for the development of hyposalivation in diabetes- induced xerostomia. Understanding the role of NOS-BH4 pathway may reveal to be promising for developing new therapies for DM patients suffering from xerostomia.

#### Bone Morphogenetic Protein 7

Bone morphogenetic protein 7 (BMP7) is a protein in the transforming growth factor β superfamily, which has a multifunctional role in the control of cell proliferation, differentiation, growth and apoptosis (Cecchi et al. 2016). It has been shown that high levels of BMP7 mRNA play a significant role in secretory and degenerative changes in salivary glands in DM mice (Izumi et al. 2008). Reduced BMP7 levels in the parotid, but not submandibular gland, were associated with DM, Table 1. Histologically, remarkable degeneration with atrophy was also found in parotid gland, while degeneration of submandibular gland was insignificant (Izumi et al. 2008). To check if this is reversible with BMP7 therapy, BMP7 (50 and 100 µg/kg, i.v.) was administered in DM mice which led to substantial increase in salivary secretion, with ratio of recovery higher in parotid gland than in submandibular gland (Izumi et al. 2008). These findings indicate that increased mRNA levels of BMP7 play a protective role in avoiding DM injury of salivary glands.

#### Common Salivary Protein 1

**Fig. 1:**
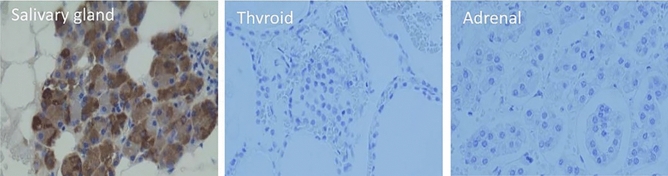
CSP1 is a salivary gland specific protein. Immunostaining of CSP1 in salivary (left), thyroid (center) and adrenal gland tissue (right) demonstrated that CSP1 stains specifically and exclusively salivary glands and not the other two tissues rendering it a salivary gland-specific protein. Wang et al., 2016. Journal of clinical laboratory analysis. Taken from: https://doi.org/10.1002/jcla.21963 (Wang et al. 2016)

Common salivary protein 1 (CSP1, LOC124220; synonyms: HRPE773, PRO1567), a salivary gland-specific protein, was notably increased in DM patients’ serum (Wang et al. 2016), Table 1. Indeed, immunostaining of CSP1 in different exocrine/endocrine tissues from healthy subjects was only detectable in salivary gland (Fig. 1). The mechanisms through which CSP1, a protein normally found in salivary secretions, translocates to the serum are unknown. The authors suggest that the same mechanisms by which serum proteins cross to the saliva could be acting in reverse. It has been shown that transport of molecules from serum to saliva occurs by transcellular (passive and active transport) and paracellular routes (ultrafiltration). Many cases involve detection of a biomarker for a specific disease in both the saliva and serum. In a recent study, the authors further investigated the salivary levels of CSP1 in DM and healthy patients. They showed a significant up-regulation in CSP1 salivary levels in DM patients, sufficient to be able to discriminate between DM and control groups (Zhang et al. 2020), Table 1. Detection of CSP1 in salivary secretions may hold a great potential as a biomarker for DM patients’ diagnosis and screening as its non-invasiveness, and consequent safety and ease of collection constitute significant advantages over blood-based diagnostics.

#### Statherin protein

Statherin is an important salivary protein in humans that is encoded by the STATH gene. It maintains the supersaturated state of saliva by supporting high calcium and phosphate concentrations. High levels of calcium promote remineralization, recalcification and stabilization of the tooth enamel and inhibit the formation of mineral accretions on tooth surfaces, while maintaining high levels of phosphate is crucial for buffering (De Sousa-Pereira et al. 2013). Statherin levels have been investigated in DM subjects in order to study its role in sustaining oral health (Izumi et al. 2015). A body of evidence indicate that statherin levels are decreased in saliva of T1DM children and in the secretory granules of parotid, submandibular and labial glands of DM patients (Isola et al. 2011b, a, 2012; Caseiro et al. 2013), Table 1. The data from those studies suggest that insufficient levels of statherin could contribute to oral cavity complications in diabetes i.e. dental caries and oral infections. This encourages further investigation about statherin’s role as a marker of salivary dysfunction which is a major problem in DM patients encounter plus overall disease activity in T2DM explicitly. Salivary statherin levels in salivary glands of DM patients could serve as screening tool for diagnosis and prognosis of DM patients.

#### Aquaporins

AQPs have been a center of investigation for their role in the pathogenesis of diabetic xerostomia (Bhattarai et al. 2017; Biswas et al. 2018; Cui et al. 2021). They are also called water channels belonging to a larger family of major intrinsic proteins (He and Yang 2019). Aquaporins facilitate the transmission of water, small solutes and gas between cells. AQP1, AQP5 and AQP8 have been studied in diabetes-induced xerostomia (Wang et al. 2011; Bhattarai et al. 2017). Three different groups of rats: control, STZ-induced DM and STZ-induced DM injected with insulin were selected in a study to show the correlation between hyposalivation in DM and the expression of aquaporins in the submandibular salivary gland in addition to the therapeutic effect of insulin (Cui et al. 2021). Immunohistochemistry tests were performed to detect AQP expression and morphological changes. Results showed a significant decrease of expression of AQP1, AQP5, AQP8 accompanied by minor degeneration at the level of acini as well as architectural changes of the acinar cells in submandibular glands, resulting in decreased salivary secretion/flow in the DM mice, Table 1. In contrast, in both control and insulin-administered DM mice, submandibular glands’ AQPs expression were increased and the morphology of the gland was intact with normal salivary secretion (Cui et al. 2021). Another study was conducted to show a therapeutic effect of a herbal medicinal extract called Ixeris dentata (IXD) on hyposalivation induced by DM (Bhattarai et al. 2017). Results showed that T1DM rats that received a single oral spray of IXD extract under the tongue had a significant increase in the expression of AQP5 in salivary gland acinar and ductal cells and consequently increased salivary gland secretion (Bhattarai et al. 2017). Effect of low-level laser therapy (LLLT) on the membrane distribution of AQP5 in xerostomia DM patients was also investigated (Biswas et al. 2018). The expression of AQP5 had been altered due to supersaturated intracellular Ca^2+^ and the ER stress caused by hyperglycemia. LLLT upregulated the membrane redistribution and expression of AQP5 and increased salivary secretion in DM rats (Biswas et al. 2018). These findings suggest that aquaporin-based therapies may play a significant role in alleviating xerostomia in DM patients.

#### Muscarinic Receptors

**Fig. 2:**
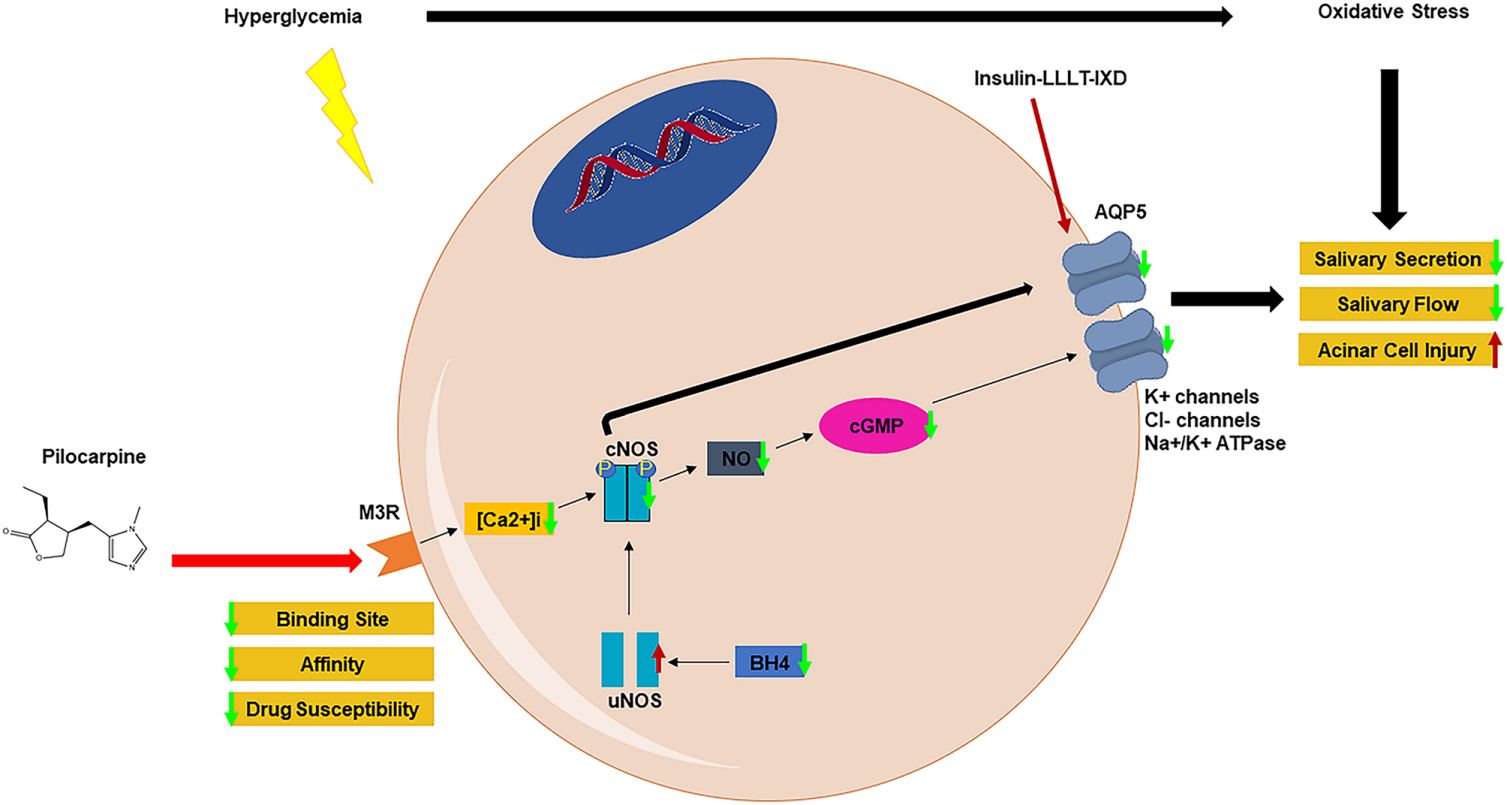
Salivary secretion pathway in DM. Consistent hyperglycemia will lead to an increased state of oxidative stress that ultimately impacts salivary secretion and flow. In diabetes, M3R’s binding sites, affinity and drug susceptibility are decreased which in turn result in a lower intracellular Ca2 + ([Ca2 +]i). Combined with a decrease in BH4, a cofactor necessary for the dimerization of uNOS into cNOS, NOS activation is decreased paralleled by a reduction in NO catalyzation and AQP5 translocation. cGMP production is decreased due to NO deficiency and fails to open ion channels (i.e. K + channels, Cl- channels, Na + /K + pumps) to initiate salivary secretion. The obstruction of this pathway in DM leads to reduced salivary flow, secretions and histomorphological changes accompanied by acinar cells degeneration. Administration of pilocarpine, a cholinergic agonist, slightly alleviates those effects, whereas insulin, LLLT and IXD work directly on recruiting and upregulating AQP5 to reverse those effects. M3R, Muscarinic M3 receptor; [Ca2 +]i, intracellular calcium; uNOS, uncoupled nitric oxide synthase; cNOS, coupled nitric oxide synthase; NO, nitric oxide; cGMP, cyclic guanylate monophosphate; AQP5, aquaporin 5; LLLT, low-level laser therapy; IXD, ixeris dentata

Saliva’s secretion and release depends on parasympathetic stimulation. A study has been conducted to demonstrate whether the decrease in salivary secretion in DM is solely due to water loss or whether it is due to a major involvement of muscarinic receptors (Watanabe et al. 2001). Saliva secretion was measured in control and STZ-induced DM rats. Under no stimulation, control and DM rats given water ad libitum manifested similar whole saliva amounts. Upon pilocarpine treatment, a muscarinic acetylcholine agonist, the secretion of saliva in both groups increased, but this increase was two- to three-fold higher in control. The same pilocarpine treatment was given to DM rats after 6 h of water restriction and this time it only slightly increased the salivary secretion in DM rats while salivary secretions were accentuated in control groups. These data indicate that salivary release in DM is gravely impacted by (presumably polyuria-induced) water loss. Pilocarpine-induced salivary release showed no dose-dependent correlation in DM rats (in contrast with controls) and in fact was significantly decreased compared to controls upon high doses indicating that DM rats have a lowered susceptibility for muscarinic receptor simulation. To further investigate the specificity and affinity of muscarinic receptors binding sites, radiolabeling assays were conducted at the level of parotid and submandibular glands. Results showed (1) a decrease in binding site of muscarinic receptor in the parotid gland membranes (2) a decrease in affinity of muscarinic receptor in the submandibular gland membranes (3) a decrease in drug susceptibility for muscarinic receptor agonists in the DM versus control rats (Watanabe et al. 2001), Table 1. The decrease in muscarinic receptor activity was accompanied by histomorphological changes and degeneration at the level of acinar cells and the authors believe that there is a direct correlation between those factors (Watanabe et al. 2001). The pathway pertaining to muscarinic receptor changes in hyperglycemic conditions and their effect on salivary secretion is illustrated in Fig. 2.

#### Heat shock protein 60 (Hsp60)

Hsp60 belongs to the molecular chaperone family, and is activated upon endogenous or exogenous stress signals to prevent cellular injury (Basset et al. 2020b). As aforementioned, DM patients present a higher risk of developing oral-related diseases, including salivary gland diseases, thus the circulating levels of Hsp60 in serum and saliva of DM patients might be expected to be elevated in response. Hsp60 levels were measured (Yuan et al. 2011) and quantification revealed a significant increase in the protein expression of Hsp60 in both the serum and saliva of DM patients compared with healthy individuals, Table 1. Hsp60’s levels in saliva were positively correlated with those in the serum, while serum Hsp60 was 16-fold higher than the ones in saliva. While the mechanism of action of the translocation of the Hsp60 in the saliva is unknown, it is speculated that this phenomenon may be attributed to their extracellular-release via the exosomal pathway. The correlation between serum and saliva Hsp60 in DM patients renders Hsp60 a candidate for biomarker studies and for screening of diabetic patients in routine monitoring.

#### Alterations of fetal salivary gland protein expression in the diabetic milieu

**Fig. 3:**
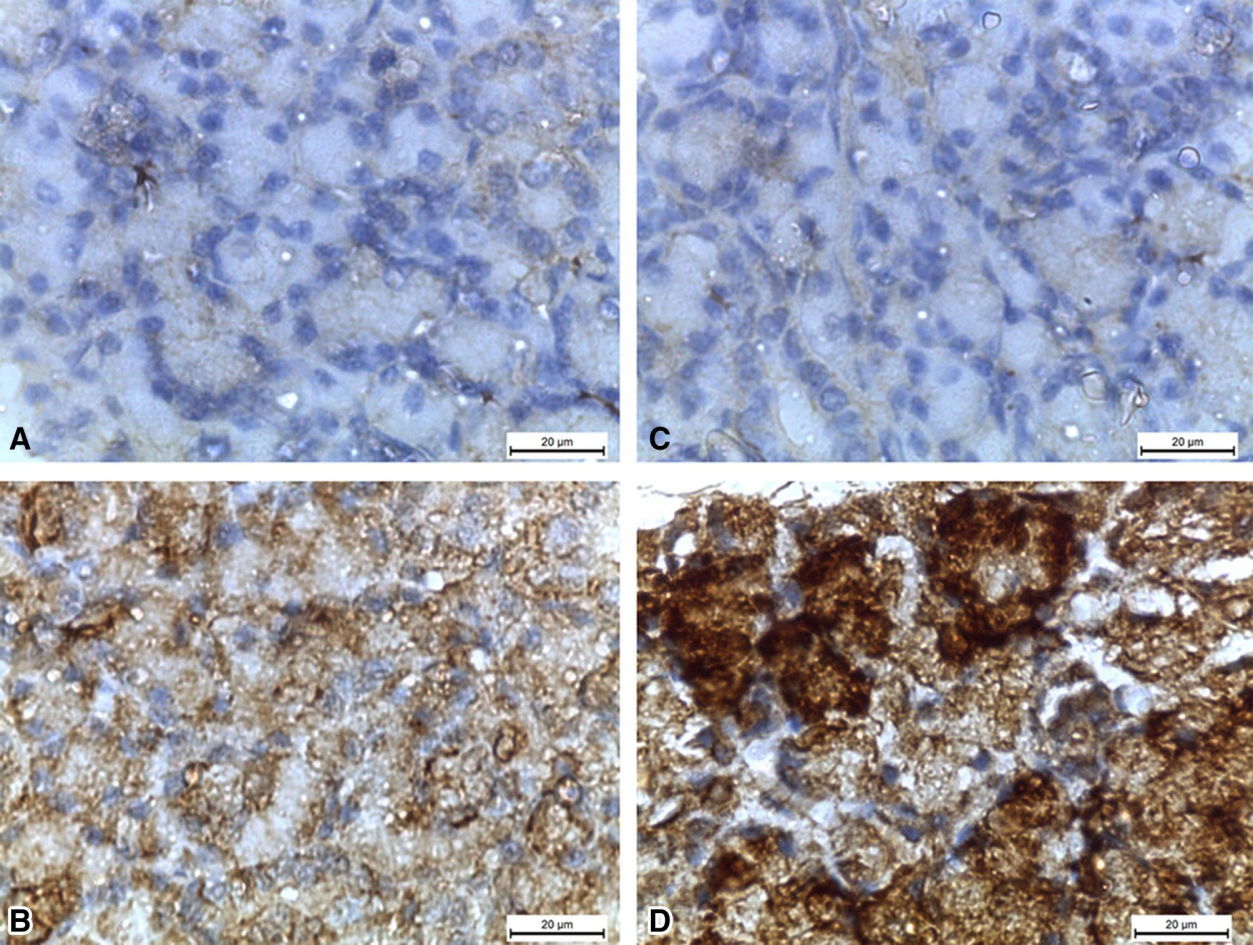
Caspace-3-mediated apoptosis increases in postnatal offspring submandibular glands of diabetic rats. **A** and **C** (2 weeks (2w) postnatal control and 4 weeks (4w) postnatal control groups) showing negative caspase-3 reaction. **B** (2w diabetic group) showing positive caspase-3 reaction in the form of brown discoloration of the cellular acini. **D** (4w diabetic group) showing very strong positive caspase-3 reaction. (Caspase-3 × 1000). El Sadik et al., 2018. PLoS One. Taken from: https://doi.org/10.1371/journal.pone.0205372 (El Sadik et al. 2018)

**Fig. 4:**
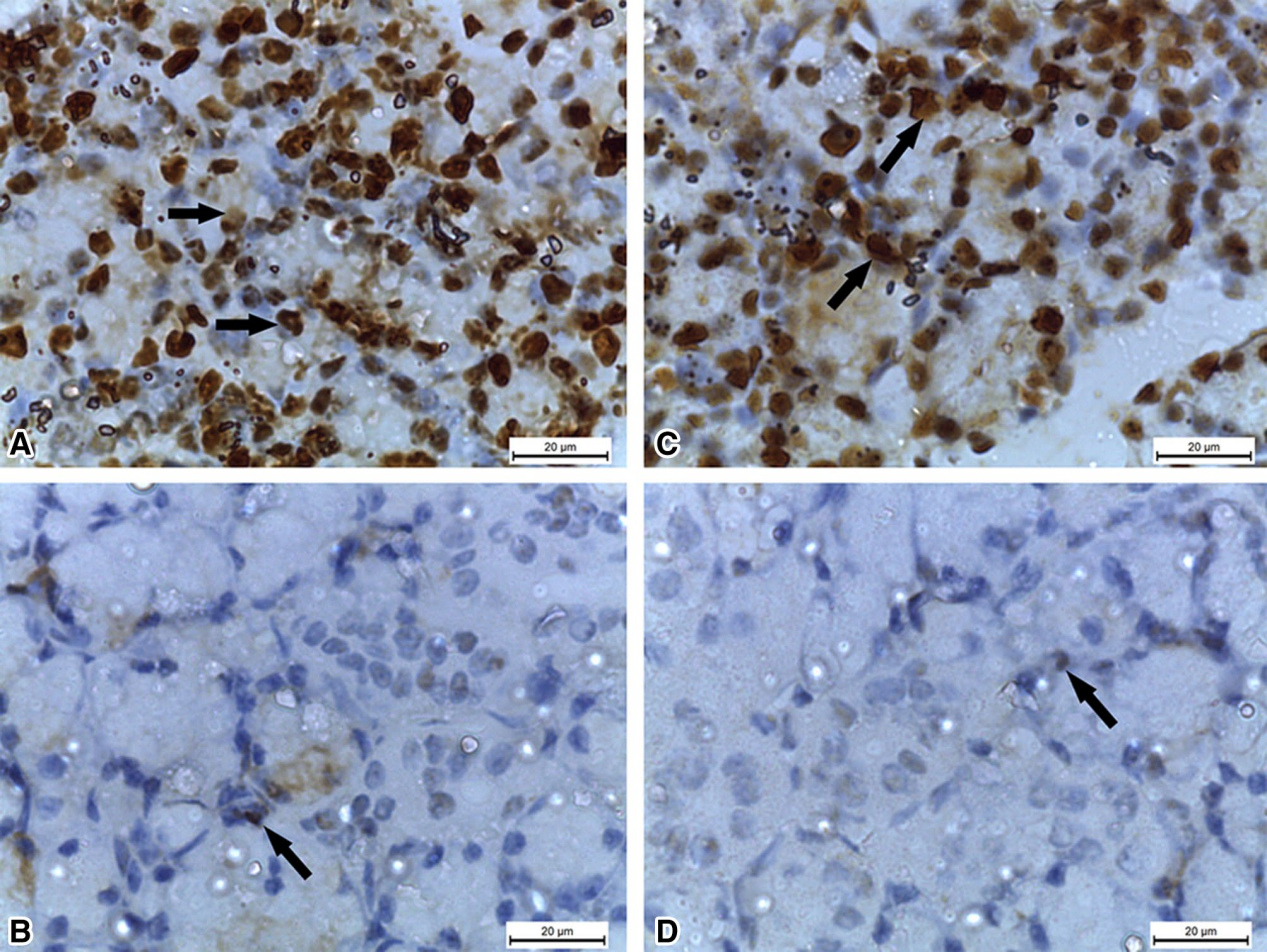
PCNA reduction impedes cell growth in postnatal offspring submandibular glands of diabetic rats. **A** and **C** (2w control and 4w control groups) showing strong positive PCNA reaction (arrows) in the cellular nuclei of the acini. **B** and **D** (2w diabetic and 4w diabetic groups) showing minimal PCNA reaction (arrows). (PCNA × 1000). El Sadik et al. 2018. PLoS One. Taken from: https://doi.org/10.1371/journal.pone.0205372 (El Sadik et al. 2018)

DM can have deleterious effects on salivary glands function in adults. A question comes to mind on whether this could be reciprocated pre-nataly where diabetic environment could modulate the physiology and morphology of the emrbyo’s salivary glands. Thus, we wanted to elaborate further on this topic as we consider it an important area of research from here on and wanted to highlight the major exacerbation of pre-natal diabetic environment on the function and architecture of emrbyonic salivary glands. Indeed, the effects of maternal DM on the submandibular glands of the off-spring rats have been investigated (El Sadik et al. 2018). DM exhibited degenerative effects in the gland morphology and function, altering their secretory activity affecting oral and digestive health. The submandibular glands of the offspring of STZ female rats were assessed at two and four weeks after birth. Several genes involved in cell growth, differentiation and cellular apoptosis were altered: epidermal growth factor (EGF), cytokeratin 5 (CK5), CK7, AQP5 and B cell lymphoma 2 (Bcl2) were downregulated; in contrast, Bcl2-associated X protein (Bax) was up-regulated (El Sadik et al. 2018), Table 1. Caspase-3 is the downstream effector of mitochondrial-cytochrome c cellular apoptosis pathway, involving Bax. Caspase-3 was reported increased along with Bax in salivary glands of the offspring of DM groups resulting in apoptosis and therefore loss of salivary gland function (Fig. 3). PCNA, a cell proliferation marker, was shown to be reduced in the salivary glands of the offspring of DM groups. Ultimately salivary gland activity was lost altogether, presumably as a result of increased apoptosis and reduced growth (Fig. 4). These results demonstrate that these genes play a significant role in saliva secretion, glands tumorigenesis, growth of normal oral flora and oral microbes, with decreased protein synthesis and production of xerostomia and dental caries. Moreover, damage of normal glandular morphology, significant increase in fibrosis and stagnation of secretory granules were found with atrophic changes in the acinar cells (El Sadik et al. 2018). Decrease in the number of secretory granules is consistent with the role of DM in reducing the human gland secretory activity. All these findings prove that intrauterine diabetic milieu affects the growth of the gland and its prenatal and postnatal architecture and function.

## Conclusion

Nowadays, studies revolving around DM acknowledge the tight association between diabetes and oral-related complications. Salivary glands dysfunction in the diabetic milieu has been a subject of interest as of late, as studies seem to suggest the importance of studying key salivary proteins in DM patients. Understanding the underlying mechanism responsible for the deregulation of salivary proteins is of crucial importance as it may be directly associated with the pathogenesis of diabetes-related salivary gland diseases. Additionally, target-based therapy of salivary specific proteins for restoring diabetes-induced salivary gland injury and degeneration should be further examined and tested. Diabetes-induced alterations of salivary proteins positively correlate with patients’ health profile. In this review, we propose that the detection of key salivary proteins may be the basis of developing non-invasive means of diagnosis and prognosis during routine screening of patients with the notion in mind that extensive research correlating salivary specific markers with serum glucose levels in DM patients is required to achieve the aforementioned results.

## Data Availability

The data that support the findings of this study are available from the corresponding author upon reasonable request.
